# FEELING VS BEING CONNECTED: DIFFERENTIATING LONELINESS AND ISOLATION IN NEAR- AND CENTENARIANS

**DOI:** 10.1093/geroni/igac059.2721

**Published:** 2022-12-20

**Authors:** Lian Ying, Chun Pat, Bobo Hi, Po Lau, Peter Martin, Joey Chung, Yue Siu, Karen Siu, Lan Cheung, Cecilia Lai, Wan Chan, Grace Man, Yee Chan, James Ka, Hay Luk

**Affiliations:** Hong Kong Shue Yan University, Hong Kong, Hong Kong; Hong Kong Shue Yan University, Hong Kong, Hong Kong; Hong Kong Shue Yan University, Hong Kong, Hong Kong; Hong Kong Shue Yan University, Hong Kong, Hong Kong; Iowa State University, Ames, Iowa, United States; Hong Kong Shue Yan University, Hong Kong, Hong Kong; Caritas Institute of Higher Education, Hong Kong, Hong Kong, Hong Kong; Hong Kong Shue Yan University, Hong Kong, Hong Kong; The University of Hong Kong, Hong Kong, Hong Kong; Hong Kong Shue Yan University, Hong Kong, Hong Kong; The University of Hong Kong, Hong Kong, Hong Kong; Hong Kong Shue Yan University, Hong Kong, Hong Kong; The Hong Kong Council of Service, Hong Kong, Hong Kong; Hong Kong Shue Yan University, Hong Kong, Hong Kong; TWGHs Fung Yiu King Hospital (FYKH), Hospital Authority, Hong Kong, Hong Kong

## Abstract

While gerotranscendence theories postulate that older adults tend to orient themselves toward solitude, activity theories highlight the importance of continuing social and meaningful engagement for well-being across lifespan. The distinction between loneliness and social isolation is particularly observable in older adults of advanced age who are often facing accelerated decline in physical and functional health, therefore restricting their opportunities to interact with others. This has been particularly disturbing during the previous two years under COVID. This study utilized data from the 2nd Hong Kong Centenarian Study which interviewed 120 family caregivers of older adults aged 95 or above in 2021–2022 when the city experienced almost an entire year of the outbreak. Using family or friend proxy information as well as caregiver ratings of whether older adults expressed feelings of social isolation and loneliness, we found that 10.7% of older adults reported high levels of loneliness and isolation; 26.7% feeling low in both; 11.5% were isolated but not lonely, and 38.2% were lonely but not isolated. Loneliness ratings were more strongly associated with psychological well-being (Patient Health Questionnaire-4), autonomy, happiness, perceived usefulness, worries, and death anxiety than did isolation, with the latter negatively correlated with optimism. Participants rated in the low isolation/loneliness group were least (death) anxious than the other three groups. Our findings underscore the divergence of isolation and loneliness for adults of advanced age and call for psychological support for oldest-old adults who continue to face social isolation, especially when society gradually recovers from COVID.

